# A Micellar-Hydrogel Nanogrid from a UV Crosslinked Inulin Derivative for the Simultaneous Delivery of Hydrophobic and Hydrophilic Drugs

**DOI:** 10.3390/pharmaceutics10030097

**Published:** 2018-07-19

**Authors:** Delia Mandracchia, Adriana Trapani, Sara Perteghella, Cinzia Di Franco, Maria Luisa Torre, Enrica Calleri, Giuseppe Tripodo

**Affiliations:** 1 Department of Pharmacy-Drug Sciences, University of Bari “Aldo Moro”, Via Orabona 4, 70125 Bari, Italy; delia.mandracchia@uniba.it (D.M.); adriana.trapani@uniba.it (A.T.); 2 Department of Drug Sciences, University of Pavia, Viale Taramelli 12, 27100 Pavia, Italy; sara.perteghella@unipv.it (S.P.); marina.torre@unipv.it (M.L.T.); enrica.calleri@unipv.it (E.C.); 3 Consiglio Nazionale delle Ricerche-Istituto di Fotonica e Nanotecnologie (CNR-IFN) Bari, Via Amendola 173, 70125 Bari, Italy; cinzia.difranco@uniba.it

**Keywords:** inulin, vitamin E, micelle, hydrogel, dual delivery, curcumin, beclometasone, drug delivery, colon delivery, combination therapy

## Abstract

Hydrogels are among the most common materials used in drug delivery, as polymeric micelles are too. They, preferentially, load hydrophilic and hydrophobic drugs, respectively. In this paper, we thought to combine the favorable behaviors of both hydrogels and polymeric micelles with the specific aim of delivering hydrophilic and hydrophobic drugs for dual delivery in combination therapy, in particular for colon drug delivery. Thus, we developed a hydrogel by UV crosslinking of a methacrylated (MA) amphiphilic derivative from inulin (INU) (as known INU is specifically degraded into the colon) and vitamin E (VITE), called INVITEMA. The methacrylated micelles were physicochemically characterized and subjected to UV irradiation to form what we called the “nanogrids”. The INVITEMA nanogrids were characterized by DSC, SEM, TEM, water uptake and beclomethasone dipropionate (BDP) release. In particular, the release of the hydrophobic drug was specifically assessed to verify that it can spread along the hydrophilic portions and, therefore, effectively released. These systems can open new pharmaceutical applications for known hydrogels or micelle systems, considering that in literature only few examples are present.

## 1. Introduction

Hydrogels are tridimensional networks formed by interconnected polymer chains to form an insoluble network. They absorb large amounts of water while still maintaining the 3D structure and the original shape while changing their volume [1]. There are several possibilities to form a polymeric network, both by chemical bond or physical interaction. Chemical hydrogels have the capability to be chemically and physically stable unless specific chemical bonds are added, or are naturally present, to allow a full or partial network degradation upon established stimuli [2]. For biomedical applications, among the most used polymers for hydrogel preparation, the great family of polysaccharides should be mentioned. Hyaluronic acid [3], chitosan and derivatives [4], dextran [5], alginate [6], cellulose [7] and heparin [8] are among the most used. Polysaccharides are also employed for colon drug delivery because most of them are specifically degraded from intestinal microflora [9,10,11]. From a pharmaceutical point of view, the polysaccharides can be considered as a gold standard in the delivery of water soluble molecules including macromolecules such as proteins and other biomolecules such as peptides or DNA/RNA fragments [12]. These kinds of molecules are mostly excluded from being delivered by other drug delivery systems (DDSs) such as those with hydrophobic groups providing a good environment for non-water-soluble molecules. Polymeric micelles are a great tool for the technologist, since they allow the drug delivery of highly hydrophobic drugs even by intravenous administration [13]. Other commonly used DDSs are undoubtedly liposomes and solid dispersions [14,15].

Polymeric micelles are self-assembling systems made from amphiphilic polymers that can carry, within their hydrophobic core, water insoluble molecules. In literature there are lots of examples that describe polymers self-assembling in micelle systems [16]. The delivery of hydrophilic drugs by micelle systems is somewhat troubling. In particular, when dual drug release is needed, for a combination therapy, choosing the right DDS would be problematic. This is the issue we will address in this paper.

The aim of this paper is to propose a DDS that can simultaneously load both hydrophilic and hydrophobic drugs. It would be accomplished by linking together the advantages of hydrogels and polymeric micelles. In theory, it could be simply accomplished by hydration of a preformed hydrogel with a micelle suspension but, in this way, the leakage of the polymeric micelles, e.g., during storage or even after administration, is predictable. Thus, we thought to provide the hydrophilic shell of a polymeric micelle with photocrosslinkable moieties to form a “nanogrid” in which the micelles represent the hydrophobic netpoints between the hydrophilic hydrogel.

The chosen polymer was inulin (INU), since it is biocompatible, nontoxic, FDA-approved for human use and, overall (for our aim), it is specifically degraded by gut microflora (*bifidobacter* degradation). INU is a hydrophilic polyfructose obtained from renewable sources widely used in the alimentary industry for its prebiotic activity (stimulating the growth of beneficial bacteria). We previously synthesized an amphiphilic derivative of INU with vitamin E (VITE) which demonstrated high self-assembling properties plus a series of features such as biocompatibility both in vitro and in vivo that makes this derivative an ideal candidate for the delivery of highly hydrophobic drugs [17]. On the other hand, INU was also proposed for the formation of pharmaceutical hydrogels that showed favorable characteristics for colon drug delivery.

Here we show how the methacrylated INVITE, named INVITEMA, can form a micellar-hydrogel nanogrid specifically designed for the delivery of hydrophilic and hydrophobic drugs into the colon.

## 2. Materials and Methods

### 2.1. Materials

Triethylamine (TEA), dicyclohexylcarbodiimide (DCC), *N*,*N*-dimethyl-formamide (DMF), d-α-tocopherol succinate, methacrylic anhydride (MA), pyrene, diethyl ether, dimethyl sulfoxide (DMSO) and dimethylsulfoxide-*d*_6_ (DMSO-*d*_6_), Methanol HPLC grade, beclomethasone dipropionate (BDP) and inulin from Dahlia tuber (INU, *M*w ~5000 Da) were from Sigma-Aldrich (Milan, Italy) and used without further purification. *N*-hydroxysulfosuccinimide sodium salt and curcumin (CUR) were from TCI (Zentek S.R.L, Milan, Italy). Dialysis membrane MWCO 3500 Da were from Spectrum Labs (VWR international, Milan, Italy). The used water was from a Milli-Q gradient A-10 system (18.2 MΩ cm, organic carbon content ≤4 μg/L) (Merck-Millipore, Milan, Italy).

### 2.2. Apparatus

Fourier Transform Infrared Spectroscopy (FTIR) spectra were registered as KBr pellets with a Perkin-Elmer 1600 IR Fourier Transform Spectrophotometer (Perkin-Elmer, Monza, Italy). The resolution was 1 cm^−1^. Recording range 4000–400 cm^−1^.

Centrifugations were performed with a Beckman Avanti 30 (Beckman, Milano, Italy) equipped with a temperature control.

UV-Vis spectra were obtained by a Perkin–Elmer Spectrometer Lambda 25 (Perkin-Elmer, Monza, Italy).

^1^H-NMR were acquired with a Varian Mercury 300 MHz instrument (Varian, Segrate, Italy).

Hydrodynamic size and polydispersity index (PDI) of INVITEMA micelles were evaluated by a Zetasizer Nano ZS (Malvern Instruments Ltd., Worcestershire, UK).

UV irradiation of INVITEMA nanomicelles was accomplished with a “Polymer” reactor (Helios-Italquartz, Milan, Italy) provided with an UV lamp of 125 W having an emission range 250–364 nm. Emission peak 310 nm.

DSC studies were from a DSC 822E Mettler-Toledo instrument (Milano, Italy) provided with a refrigerated cooling accessory.

TEM studies were performed by a Jeol-Jem-1200EXII (Tokio, Japan). In particular, a copper grid was used as support to deposit a drop (1 mg/mL) of INVITEMA suspension, after drying at 25 °C in a desiccator the grid with the deposited sample was negatively stained with uranyl acetate and analyzed.

SEM pictures were acquired by sticking the samples on a stub provided with a carbon adhesive disc and subsequently subjected to coating by a 2 nm layer of palladium by using an electron beam evaporator. The used instrument was a Sigma Zeiss (Zeiss, Milan, Italy) field emission scanning electron microscope (FE-SEM). Acceleration voltage of the SEM probing e-beam was set at 3 kV; an in-lens detector and 30 μm slit aperture were used.

HPLC studies were performed with a LC Agilent (Agilent, Milan, Italy) equipped with a quaternary pump VL1260, a 1200 series Diode Array Detector, a 1220 Series standard autosampler SL, an ELSD detector 1260 Infinity, a Lab Advisor Basic Software and using an OpenLAB CDS LC ChemStation (Agilent, Milan, Italy) on Workstation HP Compaq 6300.

### 2.3. Synthesis of INVITEMA Conjugates

The INU-α-tocopherol succinate conjugate (INVITE) was synthesized as previously reported [17]. For the synthesis of the new conjugates bearing methacrylic-UV-reactive groups on the side chain (noted as INVITEMA1; INVITEMA2 and INVITEMA3), 200 mg of INVITE were solubilized in 10 mL of anhydrous DMF and then, calculated amounts of trimethylamine (TEA) and methacrylic anhydride (MA) were added according to the following ratio being “RU” Repeating Unit:

R_1_ = Moles of MA/Moles of RU INVITE = 0.2; 0.35; 0.5

R_2_ = Moles of TEA/Moles of RU INVITE = 0.5

The reaction mixture was maintained at 25 °C for 20 h under constant stirring and under N_2_ atmosphere. Then, the product was recovered by precipitation in Et_2_O and purified by 5 washing cycles using the same solvent. After each washing, the polymer powder was recovered by centrifugation at 4 °C, 5 min, 4000 rpm. The products were obtained with an 86–98% *w*/*w* yield range and were characterized by ^1^H-NMR spectroscopy and FTIR, critical aggregation concentration (CAC) determination, TEM and SEM, dimensional analysis, and stability studies. Derivatization degree (DD mol/mol %) was calculated from ^1^H-NMR as ratio between the integral of the peaks at 5.70 and 6.08 ppm (MA, s, 2H) and the integral of the peaks at 3.5–4.0 ppm (fructose ring, m, 7H).

### 2.4. CAC Determination

CAC determination for INVITEMA conjugates was accomplished by using the pyrene method as already reported [18].

In particular, excitation was set at 334 nm and emission was registered in the wavelength range 350−450 nm. Both excitation and emission slit was fixed at 5 nm. Pyrene was added to the INVITEMA sample solutions: final concentration of pyrene 2 × 10^−6^ M. 83.4 μL of pyrene stock solution, in acetone, (6 × 10^−3^ M) was combined with water, to gain an actual pyrene concentration of 2 × 10^−5^ M. This solution was maintained under vigorous stirring and, to remove the acetone, it was maintained under continuous nitrogen flow. Different INVITEMA water solutions in the concentrations range 10^−7^−2.5 mg/mL were prepared and to 0.9 mL of these suspensions 100 μL pyrene water solution were added. This mixture was left equilibrating overnight. Finally, the ratio of the intensities I_1_ and I_3_ against INVITEMA concentration was plotted.

### 2.5. Preparation and Characterization of Empty or Drug-Loaded INVITEMA Micelles by Direct Dialysis Method

Micelles from INVITEMA3 conjugate were prepared with concentration above its critical aggregation concentration (1.2 × 10^−2^ mM). To obtain polymeric micelles, a DMSO solution of INVITEMA3 conjugate was prepared in the presence or not of 10% *w*/*w* of beclomethasone dipropionate (BDP) and dialyzed vs. distilled water for 3 days. In particular, the polymer-drug or polymer DMSO solution, was poured into a 3500 Da MWCO regenerated cellulose (RC) dialysis membrane sealed by clamping the two extremities. The filled dialysis tube was put in a 3 L vessel and left under mild stirring performing regular water changes. After three days, the micellar water dispersion was filtered by a 0.45 µm RC membrane to eliminate non-loaded drug and, subsequently, lyophilized and recovered with a 90% *w*/*w* yield.

### 2.6. Differential Scanning Calorimetry Studies

Five mg of each sample were exactly weighed. The tested temperature range was 25–250 °C for BDP and INVITEMA products setting the heating rate at 5 °C/min. For T_g_ determination, a cycle method was applied as specified: (1) heating to 160 °C (30 °C/min); (2) cooling to −20 °C (30 °C/min) (3) heating to 160 °C with (30 °C/min). Sample cells were purged with nitrogen (flow 50 mL/min). The midpoint of the deflection in the heat flow vs. temperature curve, as belonging to the second heating cycle, was considered as the T_g_. Non-hermetic aluminum pans were used, each measurement was repeated three times.

### 2.7. Size Distribution and Morphology

INVITEMA aqueous dispersions, at concentration of 1 mg/mL were allowed to equilibrate at 25 °C, overnight, under slow stirring. After this time, the micellar dispersions were filtered by a 0.45 μm filter and analyzed by the indicated instrument. The measurements were performed in triplicate for each sample.

### 2.8. Preparation of INVITEMA Nanogrids

INVITEMA (60 mg/mL) samples were dispersed in degassed Milli-Q^®^ grade water and poured in Pyrex vials. The Vials containing the micelle suspension were irradiated for 30 min, 1 h or 2 h, without any photo-initiator, see Section 2.2 for the used equipment. After the irradiation time, the obtained nanogrid was recovered and freeze-dried.

### 2.9. Water Uptake Studies

Water uptake studies were performed in water at 37 °C for 24 h. In particular, the established amount of INVITEMA nanogrids (≈30 mg) were put in a glass tube open, the used tubes are opened in the lower part and provided with a support glass filter of 10 mm in diameter and a G2 porosity. The tubes were so put in water at 37 ± 0.1 °C and the nanogrids left to swell. After each established time point, namely 1, 2, 3 and 24 h, the tubes were allowed to percolate the excess water at atmospheric pressure, then, the tubes were placed inside a centrifuge tube and centrifuged for 5 min at 3000 rpm.

Water uptake was calculated as: q = Ws/Wd, where Ws is the weight of the swelled nanogrids while Wd is the weight of the dry nanogrids.

The reported values are the mean of three measurements for each time point and are in accordance with a standard error of 5%.

### 2.10. Drug Loading Evaluation on INVITEMA3 Nanogrid

For the drug loading evaluation of INVITEMA3 samples, 10 mg of freeze-dried micelles were dispersed in 10 mL of dimethyl sulfoxide [19]. Drug loaded BDP was calculated by HPLC as specified in Section 2.12. The loaded amount of BDP was calculated by a calibration curve of BDP as specified in Section 2.12. We also attempted to load curcumin in the same way than BDP but, not surprisingly, it degraded upon UV irradiation. Each measurement has been performed in triplicate.

The drug loading (DL *w*/*w* %) in INVITEMA3 was calculated based on the following formula:

DL (*w*/*w* %) = (BDP HPLC-calculated loaded weight/weight of nanogrids plus BDP) × 100

The Encapsulation efficiency (EE%) was calculated as:

EE% = (BDP HPLC-calculated loaded weight/theoretical BDP amount) × 100

### 2.11. Evaluation of Drug Stability upon UV Irradiation

To evaluate a possible UV degradation of BDP during UV-induced crosslinking of BDP loaded INVITEMA nanomicelles, a solution of BDP in methanol or acetone at 10% *w*/*v* was prepared. This solution was irradiated in the same conditions of the INVITEMA bioconjugates (see Section 2.8), then, the solution was dried under vacuum, the drug recovered and analyzed as described in Section 2.12.

### 2.12. Drug Release Studies from INVITEMA Nanogrids

10 mg of BDP loaded INVITEMA nanogrids, were suspended in 1 mL of Milli-Q^®^ grade water and the solution poured into a dialysis membrane with a molecular weight cut-off of 3500 Da. The dialysis tube was put in a vessel with 10 mL of PBS pH 7.4/Tween 80 0.5 *w*/*w* %, at 37 °C.

At scheduled time points, namely 1, 2, 4, 8, 24, 48, 72 and 120 h, the whole release medium was collected and replaced with fresh release media at 37 °C. The amount of released BDP has been calculated by HPLC as follow: column C18 (250 × 46 mm, 5 µm, Zorbax) from Agilent (Milan, Italy), flow rate 1 mL/min, wavelength 238 nm, mobile phase MeOH/H_2_O 70:30 *v*/*v* isocratic. The calibration curve of BDP was performed by dissolving it in HPLC grade MeOH in the concentration range 0.5–150 µg/mL, at least 6 concentration points were set inside the interval and each point was performed in triplicate (R^2^ = 0.9987).

To verify the maintenance of sink conditions along the experiment, BDP dissolution was evaluated as follow: based on the calculated DL %, 100 µg of BDP were suspended in 1 mL of Milli-Q^®^ grade water and the solution poured into a dialysis membrane with a molecular weight cut-off of 3500 Da. The dialysis tube was put in a vessel with 10 mL of PBS pH 7.4/Tween 80 0.5 *w*/*w* %, at 37 °C. The experiment was carried on until the 100% drug dissolution was reached.

## 3. Results

### 3.1. Synthesis and Characterization of INVITEMA Conjugates

**Figure 1:**
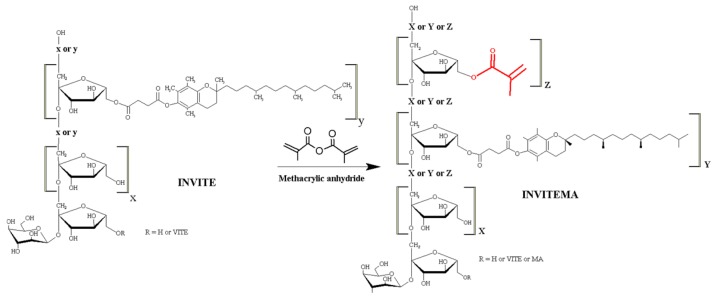
Scheme of reaction between inulin-vitamin E (INVITE) and methacrylic anhydride (MA) leading to INVITEMA conjugate.

The applied synthetic route for obtaining the INVITEMA bioconjugates proceeds through a two-step reaction (i) synthesis of vitamin E-Inulin bioconjugate INVITE and (ii) synthesis of INVITEMA, Figure 1.

The first step allowed the formation of the ester group between VITE and INU to provide the hydrophilic polymer with hydrophobic moieties leading to an amphiphilic derivative self-assembling in nanomicelles as previously described [17,19,20]. The second step introduced methacrylic moieties that undergo UV-induced radical formation on double bond leading to crosslinking of adjacent polymeric chains. Both reactions exhibited an almost quantitative yield and the actual DD% reflected the molar amount used during the reaction. INVITEMA derivatives were obtained with three different DD% based on the applied reaction conditions. As can be seen from Table 1, they were obtained with a DD% of 18, 33 or 49%. The three products at different DD% were prepared to evaluate the optimal polymer concentration during the UV irradiation [21].

### 3.2. Spectroscopy Studies, FTIR and ^1^H-NMR

The first characterizations of the INVITEMA bioconjugates were aimed at confirming the new chemical bond formation.

**Figure 2:**
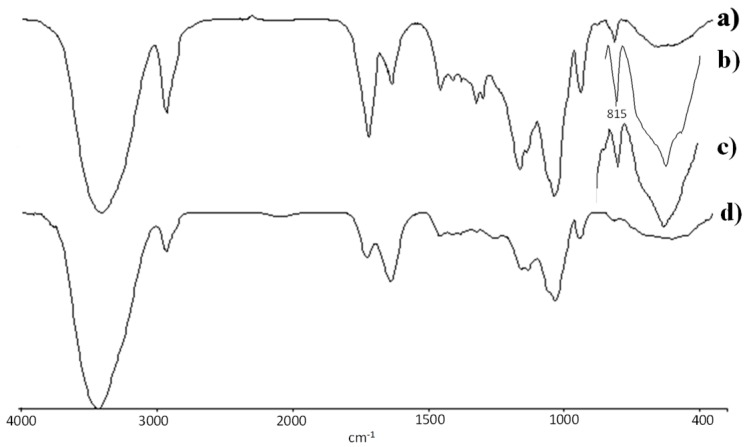
FTIR spectra of: (**a**) INVITEMA micelles; (**b**) crosslinked INVITEMA after 30 min UV irradiation still showing the presence of the acrylic double bond; (**c**) crosslinked INVITEMA system after 1 h UV irradiation still showing the presence of the acrylic double bond and (**d**) crosslinked INVITEMA system, spectra were acquired in transmittance. To improve the spectra readability only the acrylic bands at 815–816 cm^−1^ for samples irradiated at 30 min or 1 h are inserted.

FTIR spectra of the INVITEMA bioconjugates, Figure 2, showed bands from INU (1646 cm^−1^) and a new band at 1742 cm^−1^ (C=O stretching of esters). The MA linking was assessed by the presence of the bands at 1325 cm^−1^ (in-plane deformation vibration –C=C-H) and at 816 cm^−1^ belonging to the wagging of the acrylic double bond (partially overlying INU bands), spectrum “a, b and c” in Figure 2.

**Figure 3:**
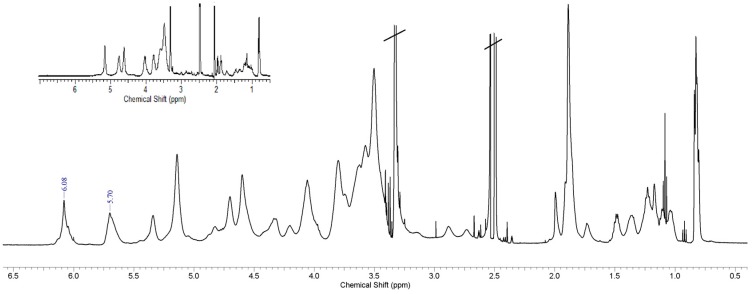
^1^H-NMR Spectra of INVITEMA conjugate in DMSO-*d*_6_, in the insert the INVITE spectrum before MA functionalization.

^1^H-NMR spectra of INVITEMA conjugates, Figure 3, showed new peaks (with respect to native INU and INVITE) at 5.70 and 6.08 ppm belonging to MA vinyl protons (2H, s, CH_2_=R), Figure 3.

### 3.3. Thermal Behaviors of INVITEMA Conjugates

The thermal behaviors of the products obtained in this work were evaluated to verify the presence of crystalline clusters within the polymeric chains and/or to verify that the entrapped (drug loaded) BDP was in the most soluble amorphous state after loading.

DSC studies were performed on INVITEMA micelles (drug loaded or not), INVITEMA nanogrids (drug loaded or not), free BDP and physical mixtures, Table 2. The study was performed uniquely on sample INVITEMA3 because, as explained in Section 3.6, it was the one giving a complete methacrylic double bonds consumption upon UV irradiation.

From DSC studies what emerges is the presence of a T_g_ for the INVITEMA micelles, this result is in agreement with our previous studies on INVITE bioconjugates [22]. Another important features of this system is that the loaded BDP stays amorphous after crosslinking and freeze drying. It can be noted that T_g_ disappears after UV irradiation up to 2 h, while is still present after UV irradiation for 30 min or 1 h. This datum is strictly connected with the (macro) molecular motion after crosslinking, as discussed below in Section 3.6.

### 3.4. CAC Determination for INVITEMA Amphiphiles

CAC for INVITEMA1, 2 and 3 bioconjugates was evaluated by the pyrene method by plotting the ratio I1/I3 versus the INVITEMA bioconjugate concentration, Table 3. The CAC values for INVITEMA1, INVITEMA2 and INVITEMA3 were estimated at the interception between (1) the rapidly varying part and (2) the nearly horizontal part at high concentration of the pyrene I1/I3 ratio plot. Results show that all the obtained bioconjugates have comparable CAC values of ≈1.2 × 10^−2^ mM. This result is in accordance with the values found for INVITE (not methacrylated) bioconjugates [17] the same reference provides the method for CAC calculation as mM and, thus, the formula used for the calculation of derivatives molecular weight.

### 3.5. Size Distribution and Stability of INVITEMA Micelles before Crosslinking

**Figure 4:**
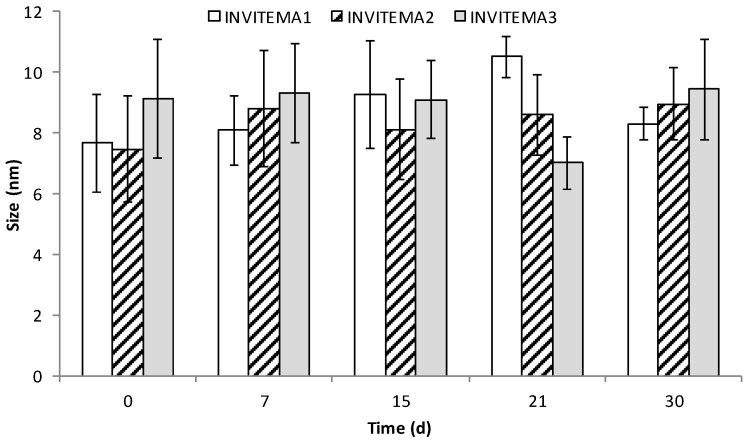
Size distribution and stability studies of INVITEMA 1, 2 and 3 micelles up to 30 days in ultrapure water by DLS.

The hydrodynamic size and the polydispersity index of the INVITEMA micelles were measured by DLS at a concentration of 1 mg/mL (above the CAC for all the samples), the gained values were: 7.67 nm (PI: 0.28) for INVITEMA1, 7.74 nm (PI: 0.31) for INVITEMA2 and 9.13 nm (PI: 0.24) for INVITEMA3, see Figure 4 time 0. Furthermore, to verify the physical stability of the micelle over the storage time in solution, the hydrodynamic size of the micelles was measured at established time points up to 30 days in water.

As from Figure 4, the micelles reached their actual hydrodynamic size immediately after reconstitution in the water medium and retained their size upon the tested time. It indicates that the micelles are stable to aggregation also assuring a good size control during UV irradiation.

### 3.6. Nanogrid Formation, SEM and TEM Analysis

Once bioconjugates and micelles characterization was accomplished, the second step of this work was to set and test the nanogrid formation. As a first set of experiments, all the INVITEMA bioconjugates (1, 2 and 3) were tested for their crosslinking behaviors. The crosslinking was monitored by FTIR and, in particular, by verifying the disappearance of the bands at 1325 cm^−1^ (in-plane deformation vibration –C=C-H) and at 816 cm^−1^ belonging to the wagging of the acrylic double bond (partially overlying INU bands), spectrum “a” in Figure 2. Spectrum “b” in Figure 2, shows the crosslinked INVITEMA3 derivative. In this spectrum, both bands at 1325 cm^−1^ and at 816 cm^−1^ disappeared thus indicating the full consumption of methacrylic double bonds during UV irradiation. By FTIR was possible to assess that only the combination INVITEMA3 and 2 h irradiation, led to full double bonds consumption. It should be underlined that all the samples and tested times gave the formation of insoluble (crosslinked) products but only INVITEMA3/2 h showed the disappearance of the acrylic bands in FTIR. Similar outcomes can be gained from DSC studies; in fact, the only sample not showing any T_g_ was the nanogrid from INVITEMA3/2 h as a result of the impeded intramolecular motion upon complete crosslinking.

**Figure 5:**
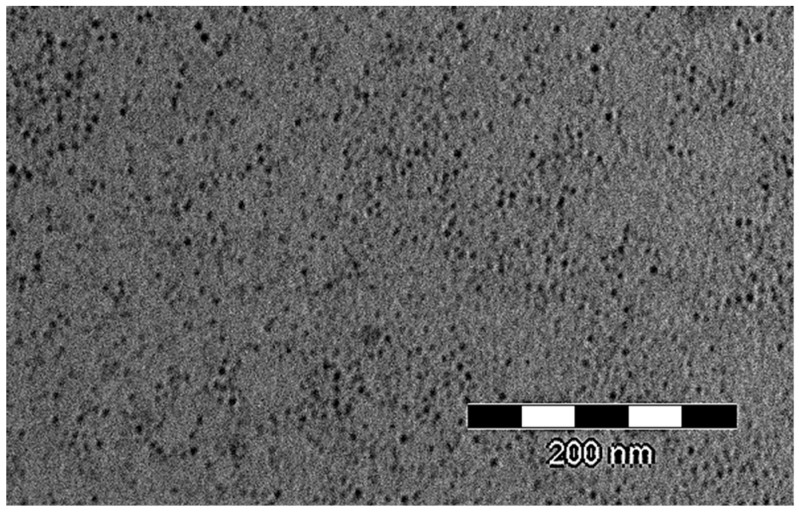
TEM image of crosslinked INVITEMA3/2 h nanogrid.

**Figure 6:**
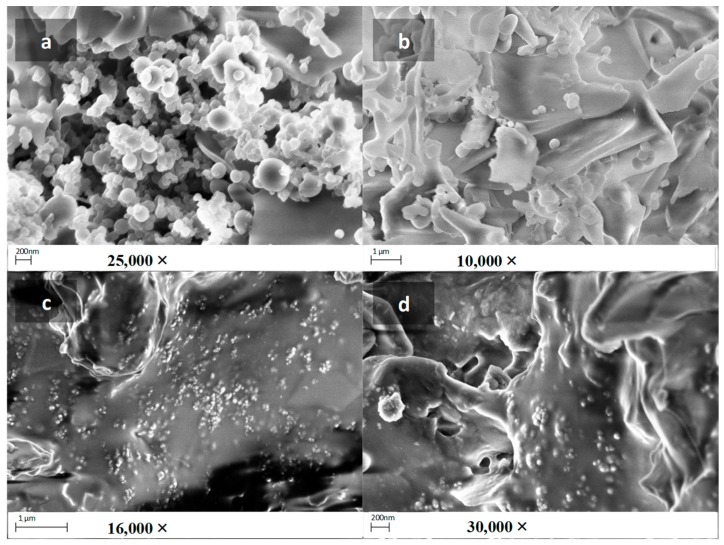
SEM images of crosslinked INVITEMA nanogrids, (**a**) INVITEMA3/30 min; (**b**) INVITEMA3/1 h; (**c**) INVITEMA3/2 h 16,000× and (**d**) INVITEMA3/2 h 30,000×.

From TEM studies, Figure 5, it was possible to see a dispersion of darker spots, corresponding to the micelles, surrounded and by a continuous matrix. From this picture is not completely clear whether the continuous matrix belongs to INVITEMA crosslinking or is the background of the copper grid used for TEM analysis. Thus, a morphological characterization was performed also by SEM, Figure 6. From these pictures it is clear the “grid-like” structure formation, in fact, dispersed in a continuous foil of the polymer, the micelles emerge clearly, c and d in Figure 6. In particular, from the picture at higher magnification (30,000×) it is clear that the nanomicelles are inside the foil and not absorbed on the surface, i.e., they are part of the material. Moreover, the random distribution of the micelles inside the material perfectly fits with that seen from TEM.

From picture a and b, in Figure 6, it is possible to note, respectively, the formation of “nanocapsules” after 30 min irradiation and the formation of the polymeric foil after 1 h.

### 3.7. Water Uptake Studies on INVITEMA3

**Figure 7:**
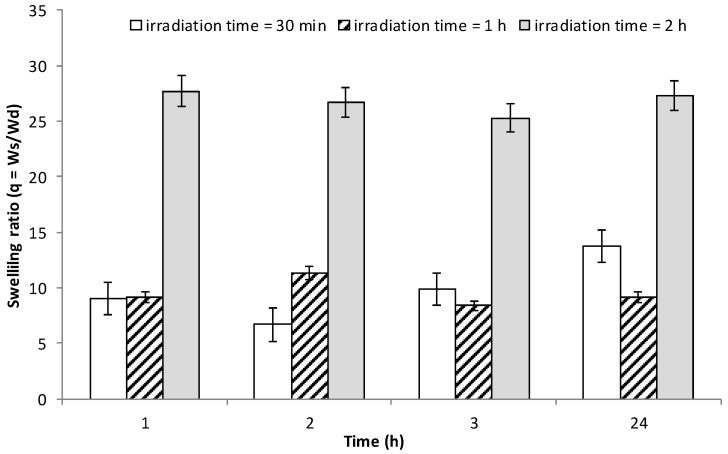
Water uptake studies on INVITEMA3 after UV irradiation for 30 min, 1 or 2 h up to 24 h in water.

Water uptake is an important feature for hydrogels. It describes how the system will behave in water and strongly influences the drug release properties of the materials. As from Figure 7, water uptake for the INVITEMA3 nanogrids is strongly influenced by the UV irradiation time, while the maximum amount of water is taken up almost immediately and remains constant up to 24 h, please refer to the discussion part. The trend seen for sample INVITEMA3/30 min could not be considered significant since within the experimental error.

### 3.8. BDP Release Studies from the INVITEMA Nanogrids

As last characterization, we performed the drug release studies of BDP. The DL% resulted 2% *w*/*w* while the EE% resulted 20%.

Drug release studies are shown in Figure 7. The amount of BDP released is expressed as percentage of released BDP with respect to the calculated drug loading. The drug release rate resulted almost constant for the whole experiment showing a controlled release due to the occurrence of both diffusion of the drug molecules through the hydrophilic layer and to the hydrophobic interaction within the hydrophobic core. Sink conditions are maintained as demonstrated by the BDP dissolution curve. BDP stability upon UV irradiation, as described in Section 2.11, did not return any variation in the elution time or area under the peak in samples at the same concentration before and after UV treatment.

## 4. Discussion

We recently developed the INVITE platform based on the conjugation of inulin and vitamin E to obtain amphiphilic polymers able to self-assemble in polymeric micelles at low concentration, showing a high biocompatibility and a tailorable chemical functionalization [23,24,25]. These systems are extremely versatile but, as with all the micelle systems, have a limitation of mostly including, within the hydrophobic core, hydrophobic molecules. While this fact is a great advantage for drugs showing low water solubility, it could constitute a problem when the delivery of a hydrophilic molecule is required, especially in combination therapy. Several examples can be found in the literature of combination therapy using micelle systems included in hydrogels [26,27,28]. Only a few examples in the literature report on the formation of hydrogels from modified micelles for drug delivery [29,30,31]. Undoubtedly, it is a great advantage to have a system formed from a hydrophilic matrix showing drug-accessible hydrophobic clusters that can allocate a wide variety of drugs.

This is what drove our work: we were looking for a drug delivery system to be proposed for colon delivery with the intention of delivering both hydrophilic and hydrophobic drugs in the course of, e.g., chronic diseases such as Crohn’s disease. In particular, we imagined a nanogrid system in which the main hydrophilic network is formed by the crosslinking of the external shell of inulin-based micelle while the hydrophobic portion is formed by the vitamin E core of the self-assembling system. Inulin is an optimal candidate for colon delivery because it is specifically degraded in the colon as result of *bifidobacter* action, INU is a prebiotic.

With this aim we functionalized INVITE micelles (INU functionalized with VITE) with methacrylic groups to form the nanogrids upon UV irradiation. This crosslinking technique was chosen since it does not require any purification step (no radical initiator was needed) and assures a good degree of sterilization. The covalent linking of the methacrylic moieties was confirmed by FTIR and ^1^H-NMR studies as discussed in Section 3.2. The self-assembling properties of the INVITEMA system were assessed by CAC studies that returned a value of 1.4 × 10^−2^ mM for INVITEMA3. This value is really low even when compared with commercially available surfactant, e.g., sodium dodecyl sulfate (SDS) shows a CMC of 7.96 mM, dodecyltrimethylammonium bromide (DTAB) shows a CAC of 13.88 mM while Tween−20 shows a CAC of 0.053 mM [17]. The achieved result from CAC suggests a good stability of the micelle system upon dilution and in the time course of UV irradiation, this assumption is further corroborated by the DLS studies that demonstrated a dimensional stability of the micelles up to 30 days.

Nanogrid-hydrogel formation took place upon UV irradiation. The consumption of methacrylic double bonds was monitored by FTIR and, in particular by the disappearance of the characteristic bands at 816 and 1350 nm representative of the double bonds. Since the radical polymerization prompted by UV is dependent on both concentration and time of irradiation, the results obtained from the three derivatives showing a different degree of derivatization at different times are extremely variable. The polymers’ concentration was constant and equal to 60 mg/mL. What mostly influenced the crosslinking was the number of formed radicals during the irradiation. The concentration of 60 mg/mL, which corresponds to 6% *w*/*v*, was chosen to maintain a low viscosity of the suspension to optimize a UV ray deep penetration. As from FTIR, only the sample INVITEMA3 after irradiation for 2 h brought the complete consumption of the methacrylic double bonds. For this reason, this sample was chosen for the subsequent characterizations. In particular, TEM and SEM studies confirmed the formation of the nanogrids, shown as spherical protuberances immersed in the polymeric foils. Their distribution appears random but the maintenance of the spherical shape and the emergence from the foil surface lets us hypothesize that upon UV irradiation the micelles first form a “nanocapsule” due to the crosslinking of the micelles shell, and then the outer methacrylic groups will react with adjacent micelles forming the polymeric foil.

This assumption is also demonstrated by the SEM pictures in Figure 6; after 30 min UV irradiation, spherical nanocapsules are mostly present, and upon increasing the time of irradiation the polymeric foil grows until a continuous layer is formed. The “nanocapsules theory” is also supported by the qualitatively evaluable size differences between the mean hydrodynamic size of the micelles before UV crosslinking that, as seen in DLS studies, results almost 10 nm and the size from the scale bar of SEM that is evaluable is almost 30 nm. This fact can be explained by considering that upon UV crosslinking the outer hydrophilic layer of the micelle can grow and retains its size after freeze drying, it being a swelled hydrogel. This fact is well explained by FTIR that shows the presence of unreacted double bonds after 30 min or 1 h UV irradiation. This assumption also justifies the increase of water uptake for the sample INVITEMA3/2 h. In fact, while the water uptake of the INVITEMA3/30 min and INVITEMA3/1 h is mostly affected by the swelling of the “nanocapsules”, sample INVITEMA3/2 h will expose to the environment a larger hydrophilic area due to the INU foils. It will lead to higher water uptake as shown in Figure 7.

**Figure 8:**
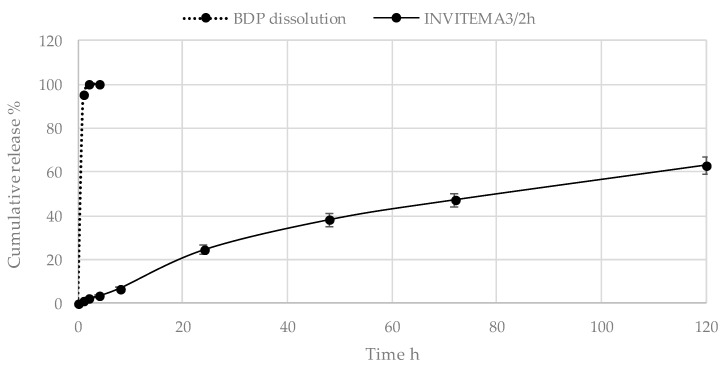
Drug release studies from INVITEMA3/2 h nanogrids, BPD dissolution curve is shown (sink conditions).

Drug release studies, as can be seen in Figure 8, show a constant release rate of the hydrophobic drug BDP. After 120 h almost 60% of the loaded drug was released. It represents the characteristic trend of the micelle system plus the effect of partitioning of the hydrophobic drug along the hydrophilic layer composed by the INU portion. In the literature, similar drug delivery systems show similar release behaviors [30,31].

## 5. Conclusions

The aim of this paper was to prepare new hydrogels provided with hydrophobic netpoints to be used for the combined delivery of both hydrophilic and hydrophobic drugs. Starting from the chemical synthesis of a methacrylated derivative of the amphiphilic bioconjugate INVITE (formed from inulin and vitamin E), we produced “nanogrids” by UV irradiation of the resulting polymeric micelle dispersion.

The obtained methacrylated derivatives were physicochemically characterized. The gained INVITEMA bioconjugates exhibited a low critical aggregation concentration that suggests a high stability of the obtained micelles upon dilution. The methacrylated micelles have been crosslinked by UV irradiation and it has been found that, depending on the irradiation time, the whole double bonds can be consumed or not. Upon complete consumption, a nanogrid was formed showing foils of hydrophilic polymer embedding the micelles, as shown by SEM analyses. Drug release studies shown a constant release rate in the tested time. We are evaluating other drugs, such as curcumin, to test the post-irradiation loading since its loading before UV irradiation results in its degradation. Biological evaluations and degradation are under investigation.

These systems are proposed for colon drug delivery due to the biodegradability of INU by intestinal microflora. Undoubtedly, the possibility of loading hydrophilic drugs such as proteic drugs together with hydrophobic drugs such as BDP provides new options for the therapy of chronic diseases such as Crohn’s disease.

## Figures and Tables

**Table 1:** Reaction conditions for the synthesis of INVITEMA conjugates, their nomenclature, molar amount used for the reaction, actual derivatization degree DD % (mol/mol) and yield % (*w*/*w*), *n* = 3.

Sample	Molar Amount of Methacrylic Anhydride (MA)	DD % mol/mol	Yield % (*w*/*w*)
INVITEMA1	0.20	18 ± 0.9	86
INVITEMA2	0.35	33 ± 1.4	93
INVITEMA3	0.50	49 ± 1.2	88

**Table 2:** Thermal behaviors of INVITEMA micelles, nanogrids (30 min, 1 and 2 h represent the different UV irradiation time), drug loaded micelles or nanogrids, beclomethasone dipropionate (BDP) and INVITEMA3/BDP physical mixture, *n* = 3.

Sample	Melting Point °C	Glass Transition (T_g_) Midpoint °C
INVITEMA3 micelles		51.4 ± 0.8
INVITEMA3 30 min nanogrid		40.1 ± 0.9
INVITEMA3 1 h nanogrid		64.6 ± 1.1
INVITEMA3 2 h nanogrid		
BDP	212 ± 0.9	
INVITEMA3 2 h/BDP physical mixture	200 ± 1.4 (BDP)	
INVITEMA3 2 h/BDP drug loaded		

**Table 3:** Found critical aggregation concentration (CAC) values, by pyrene method, for INVITEMA bioconjugates.

Sample	CAC (mM)
INVITEMA1	1.3 × 10^−2^
INVITEMA2	1.2 × 10^−2^
INVITEMA3	1.4 × 10^−2^
